# Collaborative Implementation Strategy for Newborn Resuscitation and Essential Care Training in the Dominican Republic

**DOI:** 10.3389/fpubh.2017.00061

**Published:** 2017-03-31

**Authors:** Alexandra Leader, Claudia Cadet, Davina Lazala, Wanny Roa, Olga Arroyo, Lloyd Jensen

**Affiliations:** ^1^Department of Pediatrics, Eastern Virginia Medical School, Norfolk, VA, USA; ^2^Department of Neonatology, WakeMed Health and Hospitals, Raleigh, NC, USA; ^3^North-Central Regional Health Service, Ministry of Health, Santiago, Dominican Republic; ^4^Department of Family and Community Medicine, Pontificia Universidad Católica Madre y Maestra, Santiago, Dominican Republic; ^5^Perinatal Program, Ministry of Health, Santo Domingo, Dominican Republic; ^6^Department of Pediatrics, University of Utah, Salt Lake City, UT, USA

**Keywords:** neonatal mortality, neonatal resuscitation, perinatal mortality, helping babies breathe, essential care for every baby, resource-limited settings, maternal mortality, helping mothers survive

## Abstract

**Background:**

Neonatal mortality accounts for 45% of under-5 mortality worldwide, with 98% of newborn deaths occurring in developing countries. The Dominican Republic (DR) demonstrates one of the highest neonatal mortality rates in Latin America despite broad access to care. Strategies to support professional capacity building and strengthen the local health care system are needed to improve neonatal outcomes in the DR.

**Rationale:**

Helping babies breathe (HBB) and essential care for every baby (ECEB) are evidence-based newborn resuscitation and essential care training programs that have been shown to improve providers’ confidence, knowledge, and clinical skills. Lack of professional support and infrequent resuscitation skills practice are commonly cited as barriers to skill retention after HBB training, while establishment of program mentoring and regular skills refreshers are associated with retention of clinical knowledge and skills and improved clinical performance and outcomes. Global partnerships to facilitate implementation of a comprehensive newborn resuscitation and essential care training program with ongoing clinical and program mentorship in the DR should have a lasting impact on workforce capacity, quality of care, and clinical outcomes.

**Methods:**

A multidisciplinary, international group of clinicians partnered with the Ministry of Health to design and implement a comprehensive newborn health initiative in the DR. A train-the-trainer model structured the regional rollout of a combined HBB/ECEB program with integrated quality improvement (QI) initiatives and systems for ongoing program monitoring, reinforcement, and mentorship. Cognitive, affective, behavioral, and clinical outcomes are being measured.

**Results:**

Seventeen local champions representing six hospitals participated in the HBB/ECEB master trainer course and design of a QI tool for site-specific clinical performance monitoring. One hundred seventy-eight and 171 providers participated in HBB and ECEB courses, respectively, at pilot sites during the following year. Participants completed prior training need assessment, pre-/post-knowledge assessments and course evaluations. Program mentorship and monitoring of continuing education and clinical performance are ongoing. The Ministry of Health has assumed responsibility for program sustainability and current scale-up, including integration of maternal resuscitation training.

**Conclusion:**

International partnerships facilitated the collaborative implementation of scalable, locally sustainable newborn resuscitation and essential care training in the DR, mobilizing local resources and empowering the workforce to capably pursue improved care of an exceedingly vulnerable community.

## Introduction

Neonatal mortality, defined as death within the first 28 days of life, accounts for 45% of under-5 mortality worldwide. Of the 3 million newborns that die each year, 98% of these neonatal deaths occur in developing countries, principally due to complications of prematurity, birth asphyxia, and infection (1, 2). The urgency of addressing neonatal mortality worldwide is framed by the UN’s Sustainable Development Goals (SDG 3.2) to end preventable deaths of newborns and children under 5 years of age, and specifically reduce neonatal mortality to as low as 12 per 1,000 live births in every country by 2030 (3).

The Dominican Republic (DR), which shares its western border with Haiti on the Caribbean island of Hispaniola, has an estimated population of 10.6 million people and demonstrates one of the highest neonatal mortality rates in Latin America at 22 per 1,000 live births in 2015 (4). Principal causes of neonatal mortality in the DR are low birth weight, asphyxia, and neonatal sepsis/infection, reflective of global causes of early newborn death (1). However, unlike many under-resourced countries where high neonatal mortality rates correlate with widespread lack of health-care access, the DR has a comprehensive infrastructure consisting of primary-, secondary-, and tertiary-level health-care facilities that provide strikingly inclusive coverage: 99% of all deliveries in the DR are reported to take place in health facilities and 98% of deliveries are reportedly attended by skilled birth attendants (5–7). There are numerous underlying factors likely contributing to this paradox of widespread health-care access and poor outcomes in the DR, including lack of medical equipment for newborn resuscitation and care, lack of professional supervision in clinical settings, and absence of national protocols reinforcing effective perinatal and neonatal interventions (8, 9). The Dominican National Health Plan reinforces that the *quality* of health services, rather than health coverage itself, is the challenge to improving health outcomes and relates the poor quality of care to limited resources in clinical and administrative management of the health sector, limited professional supervision, and other weaknesses of national health care infrastructure (10). These elements of the local health care system present a pivotal opportunity to reduce persistently elevated neonatal mortality by targeting sustained improvements in the quality of health care in the DR. Utilizing partnerships to support the investment of resources for newborn resuscitation and essential care and further implementing high-impact interventions to promote professional capacity building and ongoing supervision within the maternal–infant health workforce should address major limitations of the quality of health care and contribute to improved neonatal outcomes in the DR. If monitored appropriately, these predicted improvements in neonatal outcomes may provide critical evidence to champion the implementation of effective national guidelines in newborn resuscitation and care.

## Background and Rationale

Helping Babies Breathe (HBB) is an evidence-based, cost-effective neonatal resuscitation training program that has been shown to reduce neonatal mortality and improve birth attendants’ resuscitation skills in low-resource settings (11–13). Essential Care for Every Baby (ECEB) is a companion training program that reinforces basic interventions in newborn care and has been demonstrated to improve health-care providers’ confidence, knowledge, and skills related to newborn care in the first days of life in resource-limited settings (14). HBB implementation was most notably associated with an initial 47% reduction in early neonatal mortality and fresh stillborn rates in Tanzania (11). The combined implementation of HBB and ECEB in Belize was similarly associated with short-term improvement in neonatal resuscitation knowledge and skills and reduction in national neonatal mortality and stillborn rates (15).

Despite HBB’s initial success, it has also been demonstrated that providers’ resuscitation skills decline significantly over time as seen in a variety of low-resource settings in India, Kenya, and Tanzania (12, 16). Bellad et al. additionally demonstrated that rapid local scale-up of HBB training in semi-urban and rural sites in Kenya and India was not associated with consistent reduction of neonatal mortality (17). The evidence supports the need for comprehensive training and ongoing education to sustain clinical improvements. As such, a one-day HBB course in a rural Kenyan hospital did not impact course participants’ clinical resuscitation behaviors (18), whereas an initial HBB course with the complementary introduction of course refreshers and local mentorship in Tanzania was, in fact, associated with a sustained clinical impact in reduction of perinatal mortality (19). Similarly, when routine refresher training was conducted every 3 months after implementation of a neonatal resuscitation program training in Indonesia, there was observed retention of knowledge and skills of trained birth attendants 9 months after the initial training session (20). Lack of local professional support, infrequent resuscitation skills practice, and transitory nature of maternal–infant workforce are commonly cited as barriers to skill retention, while establishment of mechanisms for program mentoring and regular skills refreshers are associated with retention of clinical knowledge and skill, improved clinical performance, and improved patient outcomes (16, 21).

Sustainable improvements in maternal–infant workforce capacity and neonatal health outcomes require the development of integrated, locally sustainable training that is responsive to the specific needs of the community. The implementation of a comprehensive hospital-based newborn resuscitation and essential care training program in the DR should have a sustainable impact on the capacity of local health-care workers and thus contribute to improved quality of care and clinical outcomes if the program is founded on partnerships that facilitate continuing education and mentorship. HBB and ECEB were chosen as the optimal newborn resuscitation and essential care training programs because in addition to the evidence supporting the programs’ positive impact on knowledge, skills, and clinical performance, HBB and ECEB are disseminated using a train-the-trainer model that promotes workforce capacity by empowering local providers to be both physicians and teachers, engaging actively in the partnerships that initiate trainings and then taking responsibility for ongoing local program dissemination and clinical supervision.

What follows is a process evaluation meant to present elements of program design, content, activities, and administration of a newborn resuscitation and essential care training initiative in the DR. We will discuss the role of multidisciplinary international partnerships in bolstering the local workforce and strengthening the supply of clinical resources, leading to improvements in the quality of health infrastructure. Outcome evaluations and policy analysis based on ongoing data collection and program scale-up are forthcoming.

## Methods

This evaluation will assess the design and implementation of a neonatal health intervention composed of dual HBB and ECEB training, provision of clinical neonatal resuscitation equipment, introduction of quality improvement (QI) monitoring, and establishment of a mentoring framework to support ongoing education in the northern region of the DR.

### Site Description

The newborn resuscitation and essential care initiative have been implemented in the northern Cibao region of the Dominican Republic, in the provinces of Santiago, Espaillat, Puerto Plata, and Monte Cristi. These provinces correspond with Health Region II and part of VII (Monte Cristi) and demonstrate neonatal mortality rates comparable to the national rate of 22 per 1,000 live births, with the notable exception of Monte Cristi, where recent national statistics report a neonatal mortality rate of 31 per 1,000 live births (7). The Cibao region was chosen as the rollout site for the current initiative in the setting of ongoing successful international partnerships in various hospitals in Santiago, which is the second largest city in the DR and the capital of the Santiago Province, in addition to being the principal city in the Cibao region. Prior international collaborations in the region were redirected to focus specifically on newborn interventions at the request of hospital leadership, epidemiologist, providers, and trainees, all who feel the persistently high rates of neonatal mortality to be a call to action.

### Partnerships

A multidisciplinary group of Dominican and US-based clinicians and public health advocates, collectively representing the Ministry of Health, the Dominican Pediatric Society, the Pan American Health Organization, UNICEF, multiple international and Dominican NGOs, and individuals from three different US medical institutions met to discuss the design, implementation, and timing of the newborn health initiative. This multidisciplinary partnership represents many collective decades of clinical work, collaborative training interventions, and health promotion projects in the DR; it bears mentioning, however, that the impetus for the abovementioned stakeholders to partner for this specific project was the vital effort of one of the clinicians to communicate with other groups committed to newborn health initiatives in the DR and so prevent isolating global health efforts in the same geographic region. This early communication has been essential to the strength and success of the international partnership underlying the current newborn health initiative.

### Pilot Site Selection

Extremely high rates of mortality noted in neonates transferred to the tertiary-level Arturo Grullón Children’s Hospital in Santiago in 2013 led to the choice of five secondary-level centers within the catchment area of the Children’s Hospital as pilot sites for the newborn resuscitation and essential care intervention. In the DR, secondary health centers offer emergency, outpatient, inpatient, and basic medical specialty care services, while tertiary centers offer emergency, outpatient, inpatient, and sub-specialty services, the most advanced level of care available in the country. Four of the five intervention facilities are regional secondary-level hospitals in the provincial capitals of Santiago, Moca, Puerto Plata, and Monte Cristi, representing a significant percentage of deliveries/births in these provinces. The fifth intervention facility is an additional secondary-level teaching hospital in Santiago. Needs assessments and feasibility analyses were conducted prior to definitive site selection at the five intervention facilities to assess each hospital leadership team’s interest in the initiative, commitment to ongoing training and QI monitoring, available clinical resources, and specific barriers to newborn resuscitation and care at each site. It was during these early assessments that the process was initiated to identify multidisciplinary local champions to lead the newborn initiative at each site.

### Program Interventions

The newborn health initiative in the DR is composed of four major components: HBB and ECEB training, provision of clinical neonatal resuscitation equipment, introduction of QI monitoring, and establishment of a mentoring and supervision framework to support continuing education in the target population in the DR. The specific content and activities associated with each of these program components will be discussed in detail.

### HBB and ECEB Training

A train-the-trainer model was used for program implementation and dissemination with the goal of ultimately training all health care providers involved in newborn resuscitation and essential care partnership at each intervention site. A 4-day HBB/ECEB master trainer (MT) course was conducted in Santiago for the identified local champions from each of the five pilot intervention sites, in addition to two local champions identified at the tertiary-level Children’s Hospital in Santiago. The course was facilitated in Spanish by a multidisciplinary team of US-based clinicians with experience in HBB/ECEB and many years working in health promotion projects in the DR. Participant to facilitator ratio was 6:1. Members of the national and regional Ministry of Health services were present throughout the training. The 4-day session was divided as follows: 1.5 days for the HBB MT course, 1.5 days for the ECEB MT course, and 1 day for planning of HBB and ECEB provider courses at intervention sites, introduction of QI principles, and development of a site-specific QI tool (Data Sheet S1 in Supplementary Material).

For the planning of future HBB/ECEB provider courses at intervention sites, MTs were prompted to divide into groups according to intervention site and formulate an “Action Plan” (a format utilized in the HBB/ECEB curricula) for course dissemination, with the goal of teaching their first courses within 3 months of the MT training in Santiago. MTs were given several prompts to formulate the action plan, including questions regarding which clinical indices would be tracked to measure change (Data Sheet S2 in Supplementary Material).

The MTs subsequently returned to their health facilities and co-facilitated HBB/ECEB provider courses. The initial provider courses at three of five intervention sites were co-taught with international partners utilizing the format of 2-day sessions for the combined HBB/ECEB courses. The MTs at one intervention site in Santiago found it more feasible to structure their initial provider courses to be conducted in 2-h segments over 2 weeks and the second HBB/ECEB trainings in 3-h segments over 7 days.

### Provision of Clinical Neonatal Resuscitation Equipment

In addition to several sets of training materials for teaching HBB and ECEB courses, including multiple NeoNatalie manikins and two MamaBreast breastfeeding/manual expression simulators, each intervention site was additionally given a number of neonatal bag-mask ventilators and suction devices for clinical use in the delivery room, an attempt to address one of the identified factors underlying poor quality of care in the DR. Included in the HBB/ECEB training materials is information regarding reprocessing of clinical equipment.

### Introduction of QI Monitoring

The purpose of the last day of training during the MT course was to prepare the local champions for future dissemination of HBB/ECEB training with a focus on behavioral change in the clinical setting. Based on the content of the course material, the MTs were prompted to determine which clinical practices would be most challenging to consistently perform and which clinical parameters most important to measure (Data Sheet S2 in Supplementary Material). The MTs from each intervention site ultimately decided on a similar set of clinical parameters, including temperature at 1 h of life, skin-to-skin positioning initiated within the first hour of life, and essential care including eye care, cord care, and vitamin K administration. A QI tool was designed according to the MTs input (Data Sheet S1 in Supplementary Material) and was distributed in the form of a book of identical checklists to local champions at each intervention site for integration into the clinical setting and monitoring of clinical practices during the first hours of life.

### Data Collection

Demographic data and assessment of prior training experience, clinical roles, and nature of clinical practice are collected from course participants in the form of a questionnaire prior to the HBB/ECEB MT and provider courses. Data for knowledge outcomes are collected prior to and following both training courses in the form of standardized pre/post multiple-choice knowledge assessments published by the American Academy of Pediatrics for HBB and ECEB. Program-specific evaluations for affective outcomes related to participants’ reception of the HBB/ECEB courses and changes in attitude and confidence related to course material are collected in the form of Likert scale and free response survey questions following each pair of courses.

QI data collection is being conducted at each of the five intervention sites in the form of the QI book checklists (Data Sheet S1 in Supplementary Material), measuring the aforementioned variables of clinical practice related to newborn resuscitation and essential care.

There is additionally clinical data collection being conducted at the Children’s Hospital in Santiago. Clinical indices and care requirements of all newborns referred to the hospital (approximately 900 per year) are measured, including reason for patient transfer, vital signs and clinical status on arrival to Emergency Department, requirement for procedural interventions during first 48 h of hospitalization, and 30-day mortality. These data will allow the research team to evaluate clinical stability and survival of newborns transferred from study intervention sites as opposed to those referred from other primary and secondary centers that have not received HBB/ECEB training.

### Ethical Considerations

The American Academy of Pediatrics’ HBB and ECEB training programs are founded on global standards of care for the resuscitation and early care of newborns. The study protocol for this initiative was submitted and approved by the Institutional Review Board of Eastern Virginia Medical School. The study protocol was also reviewed and approved by the national Perinatal Program and North-Central Regional Health Service of the DR Ministry of Health. The study was additionally reviewed and approved by hospital leadership at all intervention sites, as well as the hospital leadership, residency leadership, research committee, and regional epidemiologist at the Arturo Grullón Children’s Hospital in Santiago, DR.

## Results

Seventeen local champions representing five secondary-level intervention sites and the tertiary-level Children’s Hospital participated in the HBB/ECEB MT course and design of a QI tool for site-specific clinical performance monitoring. The group of MTs included nurses, physicians, and medical technicians, all of whom participate in newborn resuscitation and essential care in their health facilities.

**Figure 1:**
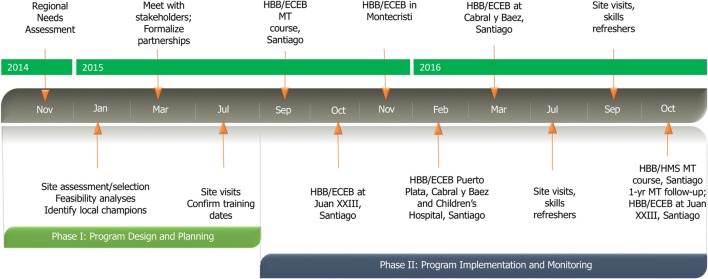
**Program implementation timeline**.

Over the course of the following year, 178 and 171 providers participated in HBB and ECEB courses, respectively, at four of five intervention sites and within the pediatric residency program at the Children’s Hospital (Figure 1). Participants in provider courses represented pediatrics, obstetrics/gynecology, and family medicine specialties, all of whom participate in newborn resuscitation and essential newborn care. A variety of training formats was utilized depending on needs and workforce availability of the intervention site. One of the five intervention sites was unable to conduct any provider courses during the 12-month period following the MT course despite ongoing mentoring from international and regional partners. All course participants completed previously described questionnaires assessing prior training and current clinical practice, pre-/post-knowledge assessments, and course evaluations at each course. International and regional program mentorship and monitoring of continuing education and program-related clinical performance parameters are ongoing.

### Establishment of Mentoring Framework

During the 12 months following the initial MT course, a member of the international training partnership was present in-country every 2 months to assist with implementation of provider courses and skills refreshers at intervention sites, troubleshoot challenges in course dissemination and implementation of QI data collection, and maintain close contact with the regional Ministry of Health and hospital leadership at each intervention site. Local champions maintain frequent communication through e-mail and WhatsApp messaging to report successes and request guidance in program dissemination and programming skills refresher sessions.

Selected local champions have assumed the role of regional mentors in addition to program leaders in their home facilities, offering to facilitate skills refreshers and co-facilitate HBB/ECEB courses with partners at other intervention sites.

The two MTs from the Children’s hospital, pediatric trainees whose residency is affiliated with the local university, *Pontificia Universidad Católica Madre y Maestra*, will focus their academic theses on issues related to the regional implementation of the newborn initiative with mentorship through international partnerships facilitating the program. As the initiative is scaled up and the QI monitoring ongoing, there are great opportunities for the research team to grow with the addition of more local trainees and ongoing mentoring from a committed multidisciplinary and international team.

### Local Sustainability

The Ministry of Health has provided consistent support of training implementation throughout the intervention sites. One year after initial course rollout, the North-Central Regional Health Service appointed a Director of Pediatric Program Dissemination to supervise further regional implementation of HBB/ECEB programs, assuming full responsibility for newborn resuscitation and essential care program sustainability and scale-up in the region.

## Discussion

In the DR, where broad perinatal health-care coverage is paradoxically associated with unacceptably high rates of neonatal mortality, there is urgency to address the availability of neonatal resuscitation supplies, professional capacity building, and clinical supervision in order to promote the quality of care delivered in the country’s health facilities. High-impact interventions to address these identified gaps in care can effectively build local workforce capacity and acquire sustainability when implemented through partnerships that support the provision of clinical and training equipment as well as empower local champions to assume responsibility for health promotion initiatives, training of their peers, and improvement in patient outcomes. HBB and ECEB are evidence-based, cost-effective newborn resuscitation and essential care training programs that have been demonstrated to build capacity for improved quality of care in newborn providers in resource-limited settings when program implementation includes continuing education and mentorship.

Strong international partnerships with a focus on collaborative training implementation facilitated the successful regional implementation of HBB and ECEB programs and initiation of QI monitoring in the northern Cibao region of the DR. The foundations for program implementation began with regional hospitals’ needs assessments and feasibility analyses at each potential intervention site and identification of local champions as early as 10 months prior to the first MT course (Figure 1). Meeting with program stakeholders to discuss design, content, and timing of regional newborn initiative 6 months prior to program rollout was decisive in substantiating the international partnerships and the Ministry of Health’s commitment to the program, which were both critical to initial program success and potential sustainability. The regional Ministry of Health’s appointment of a Director of Pediatric Program Dissemination to supervise the newborn health program sustainability and scale-up is perhaps one of the most powerful outcomes of the international partnerships and mentoring that have driven this initiative and are now supporting the reinforcement of improved quality of newborn care within the local health infrastructure.

Over the course of 13 months, after the initial HBB/ECEB MT course in Santiago, local MTs taught HBB and ECEB courses to 178 and 171 newborn providers, respectively, in five hospital settings. HBB/ECEB courses were disseminated in four of five planned intervention sites. Fidelity to training course material was seen to be possible in a variety of formats depending on the needs and availability of the workforce at each intervention site. A QI tool was designed and implemented in intervention sites by local MTs, allowing clinical behaviors outcome monitoring in addition to the cognitive and affective outcomes that are being measured through pre-/post-knowledge assessments and HBB/ECEB course evaluations, respectively, and the health status outcomes, which will be measured by regional neonatal mortality data. These data are currently being collected at all pilot sites for outcomes analysis such that the impact of the intervention will be comprehensively assessed, allowing the research team to better identify persistent gaps in training or clinical practice in order to adjust and improve the components of the intervention and strategically direct next steps of the initiative. Additionally, the data collection system established at the regional pediatric referral center will facilitate evaluation of program impact on neonatal transport outcomes. Assessing the motive for patient transfer, the vital signs and clinical status of the neonatal on arrival to the referral center, the short-term clinical interventions required after transfer, and 30-day mortality, we may indirectly evaluate the success of neonatal stabilization prior to transport, as is addressed in the ECEB curriculum.

### Challenges

Despite consistent support from the Ministry of Health, thoughtful site selection and local champion identification, and strong international and multidisciplinary partnerships invested in program implementation and mentorship, there are a number of challenges that impeded the program’s full potential progress. One of the five intervention sites was not able to conduct a provider HBB/ECEB course during the 12 months following the MT course, in spite of mentorship from international and regional partners and three site-specific MTs. Transitory nature of workforce at this site and waning motivation on the part of the MT who had been chosen to be the site representative for trainings may have determined these circumstances, although it is also worth noting that this particular site did not send the originally identified local champions to participate in the MT course, making an argument for the importance of early and consistent identification of local champions/MTs.

It has been challenging to maintain consistent QI monitoring in the intervention sites, despite the user-friendly nature of the checklist whose content was determined largely by the MTs themselves. Even when the QI book is found to be easily accessible within the delivery room of an intervention site, it is common for the providers to inconsistently enter the clinical practice data for each delivery. Only one of the five sites consistently records data at each delivery, which will be a limitation for early outcomes analysis. While mentoring seemed to be largely effective for programming of provider courses and continuing education sessions, it would appear that consistent local supervision of routine QI monitoring is needed. With a local Ministry of Health representative now responsible for program implementation and sustainability, there may be a more effective form of QI monitoring supervision available.

### Next Steps

An outcome evaluation of the newborn resuscitation and essential care initiative will be conducted through analysis of program data that is currently being collected. Continuing education in the form of additional HBB/ECEB provider courses at intervention sites and more structured skills refreshers is a priority and will be facilitated by local Ministry of Health supervision. Expansion of intervention sites is warranted and there is interest among local MTs to implement trainings in other secondary-level hospital centers, as well as the primary-level centers where deliveries are also attended.

The community needs of each intervention site drive the specific focus of training beyond the continuing education of HBB/ECEB curricula and QI monitoring. Two HBB/ECEB intervention sites have focused on utilizing ECEB program materials (MamaBreast simulator) to strengthen breastfeeding education and skills training programs for medical providers and patients. One of the sites has incorporated the breastfeeding education component into a prenatal education waiting room module in the obstetric clinic and the other site is planning a more comprehensive breastfeeding promotion project involving regular hospital-based provider and patient training and community breastfeeding support groups.

In addition to planned reinforcement and scale-up of combined HBB/ECEB throughout hospital facilities in the Cibao region, and ultimately on a national scale, steps have also been taken to make the scope of resuscitation training more comprehensive. In an effort to strengthen the continuum of maternal–infant health in the DR, where the maternal mortality ratio (92 per 100,000 births) is one of the highest in Latin America (22), a maternal resuscitation training program to reduce mortality associated with post-partum hemorrhage has been introduced alongside the newborn resuscitation and essential care programs. Helping Mothers Survive (HMS), a simulation-based maternal resuscitation training program aimed at managing obstetric emergencies in low-resource settings, was implemented alongside HBB in correlation with the one-year follow-up of the initial HBB/ECEB program rollout in Santiago. A 2-day HBB MT course was co-facilitated with six local MTs from the prior year and a multidisciplinary team of US-based clinicians to 33 physicians and nurses from 17 hospital facilities in the Cibao region. At the conclusion of the second day of HBB training, the local MT co-facilitators shared experiences, challenges, and lessons learned related to implementing provider courses in their home facilities during the first year following HBB/ECEB MT certification. This experience solidified the original MTs role as regional mentors to the newly trained HBB MTs. The HBB MT course was followed by a 2-day HMS MT course co-facilitated by a previously trained local MT and a multidisciplinary team of US-based clinicians to a group of 29 course participants from the same 17 hospital facilities in the Cibao region. Participants’ prior training experiences and clinical roles were assessed, in addition to assessment of cognitive outcomes with standardized multiple-choice pre-/post-tests and affective outcomes with HMS course evaluations. The research team, representing multidisciplinary international partners, is in the process of adapting a monitoring system to record clinical behavior outcomes in the hospital sites where HMS will be implemented. The Ministry of Health is already working with its local and global partners to form a regional HMS training team responsible for co-facilitating HMS courses with MTs in the 17 target hospitals, immediately assuming responsibility for workforce capacity building and sustainability of programs.

## Conclusion

International, multidisciplinary partnerships, and in particular, close collaboration with the Ministry of Health, facilitated the regional implementation of newborn resuscitation and essential care programs in the DR, reinforcing workforce capacity and developing local infrastructure for program sustainability and scale-up. The power of identifying and unifying the efforts of various global health teams committed to similar projects within the same region cannot be understated. Use of a train-the-trainer model with subsequent international and regional mentoring of local champions and academic trainees has contributed to capacity building of local providers, establishment of local systems of mentoring and clinical supervision, first-year program sustainability, and individualized program expansion according to particular community needs, but was not sufficient to achieve consistent QI monitoring at all intervention sites. Designation of a local Ministry of Health representative to supervise program sustainability and growth will provide greater support for regional monitoring of clinical behavior outcomes. The establishment of a strong regional framework for newborn survival training and mentorship in the northern DR facilitated the subsequent coherent integration of maternal resuscitation training on a regional scale with corresponding development of a regional maternal training team to further enhance workforce capacity and strengthen local health care infrastructure.

Partnerships that foster collaborative training interventions may contribute essential clinical resources to local providers, empower providers to better care for their patients and promote the training and supervision of their colleagues, and strengthen the local health care system, ultimately improving the quality of care, supporting improved health outcomes, and upholding the dignity of all.

## Author Contributions

AL wrote the first draft; AL, LJ, and CC contributed revisions; and all the authors reviewed the final version, provided input into the research and program design, participated in data acquisition, and approved the final manuscript.

## Conflict of Interest Statement

The authors declare that the research was conducted in the absence of any commercial or financial relationships that could be construed as a potential conflict of interest.

## Abbreviations

DR, Dominican Republic; HBB, helping babies breathe; ECEB, essential care for every baby; MT, master trainer; QI, quality improvement; HMS, helping mothers survive.
