# Supplementary material for: Collaborative Implementation Strategy for Newborn Resuscitation and Essential Care Training in the Dominican Republic
Source: Front Public Health. 2017 Mar 31;5:61. doi: 10.3389/fpubh.2017.00061 (PMC5374151; doi:10.3389/fpubh.2017.00061)
Supplement: Supplementary file 1 [file Data_Sheet_1.PDF]

Supplementary Material

Collaborative Implementation and Sustainability of Newborn Resuscitation and Essential Care Training in the Dominican Republic

Alexandra Leader*, Claudia Cadet, Davina Lazala, Wanny Roa, Olga Arroyo, and Lloyd Jensen

*** Correspondence:** Alexandra Leader: alexandra.leader@chkd.org

Figure 3: ECEB Quality Improvement Monitoring Tool

1. ID (baby's initials) ______________

2. Weight ______________

3. Temperature (at 1 hour of life): ______________

4. Did the baby need ventilation? Yes☐ No☐

5. Skin-to-skin during 1st hour of life? Yes☐ No☐

6. Did the baby receive eye care? Yes☐ No☐

7. Did the baby receive cord care? Yes☐ No☐

8. Did the baby receive vitamin K? Yes☐ No☐

10. Did you participate in HBB/ECEB training? Yes☐ No☐

11. What is your clinical role? (nurse, doctor, etc.) ______________

12. Did the baby need to be transferred to another facility for advanced care? Yes☐ No☐
