# Supplementary material for: Collaborative Implementation Strategy for Newborn Resuscitation and Essential Care Training in the Dominican Republic
Source: Front Public Health. 2017 Mar 31;5:61. doi: 10.3389/fpubh.2017.00061 (PMC5374151; doi:10.3389/fpubh.2017.00061)
Supplement: Supplementary file 2 [file Data_Sheet_2.PDF]

Supplementary Material

Collaborative Implementation and Sustainability of Newborn Resuscitation and Essential Care Training in the Dominican Republic

Alexandra Leader*, Claudia Cadet, Davina Lazala, Wanny Roa, Olga Arroyo, and Lloyd Jensen

*** Correspondence:** Alexandra Leader: alexandra.leader@chkd.org

Figure 2: Quality Improvement Action Plan

1. **Who will you train? (physicians, nurses, students, etc)**

2. **How will you get permission for trainings from hospital leadership?**

3. **Will there be a Master trainers' representative?**
   a. Form a committee?
      i. Institutional?
      ii. Regional?

4. **When will you conduct the first provider course?**
   a. Preparation steps:

5. **Identify clinical indices of change that you will monitor:**
   a. Resuscitation skills
   b. Skin-to-skin
   c. Cord care/eye care
   d. Infant temperature
   e. Vitamin K administration
   f. Antibiotics administration prior to transport
   g. Breastfeeding
   h. Parental guidance prior to discharge
   i. Referral process
